# Knowledge-Driven Analysis Identifies a Gene–Gene Interaction Affecting High-Density Lipoprotein Cholesterol Levels in Multi-Ethnic Populations

**DOI:** 10.1371/journal.pgen.1002714

**Published:** 2012-05-24

**Authors:** Li Ma, Ariel Brautbar, Eric Boerwinkle, Charles F. Sing, Andrew G. Clark, Alon Keinan

**Affiliations:** 1 Department of Biological Statistics and Computational Biology, Cornell University, Ithaca, New York, United States of America; 2 Section of Cardiovascular Research, Department of Medicine, Baylor College of Medicine, Houston, Texas, United States of America; 3 Human Genetics Center, Health Science Center, University of Texas, Houston, Texas, United States of America; 4 Department of Human Genetics, School of Medicine, University of Michigan, Ann Arbor, Michigan, United States of America; 5 Department of Molecular Biology and Genetics, Cornell University, Ithaca, New York, United States of America; Vanderbilt University, United States of America

## Abstract

Total cholesterol, low-density lipoprotein cholesterol, triglyceride, and high-density lipoprotein cholesterol (HDL-C) levels are among the most important risk factors for coronary artery disease. We tested for gene–gene interactions affecting the level of these four lipids based on prior knowledge of established genome-wide association study (GWAS) hits, protein–protein interactions, and pathway information. Using genotype data from 9,713 European Americans from the Atherosclerosis Risk in Communities (ARIC) study, we identified an interaction between *HMGCR* and a locus near *LIPC* in their effect on HDL-C levels (Bonferroni corrected *P*_c_ = 0.002). Using an adaptive locus-based validation procedure, we successfully validated this gene–gene interaction in the European American cohorts from the Framingham Heart Study (*P*_c_ = 0.002) and the Multi-Ethnic Study of Atherosclerosis (MESA; *P*_c_ = 0.006). The interaction between these two loci is also significant in the African American sample from ARIC (*P*_c_ = 0.004) and in the Hispanic American sample from MESA (*P*_c_ = 0.04). Both *HMGCR* and *LIPC* are involved in the metabolism of lipids, and genome-wide association studies have previously identified *LIPC* as associated with levels of HDL-C. However, the effect on HDL-C of the novel gene–gene interaction reported here is twice as pronounced as that predicted by the sum of the marginal effects of the two loci. In conclusion, based on a knowledge-driven analysis of epistasis, together with a new locus-based validation method, we successfully identified and validated an interaction affecting a complex trait in multi-ethnic populations.

## Author Summary

Genome-wide association studies (GWAS) have identified many loci associated with complex human traits or diseases. However, the fraction of heritable variation explained by these loci is often relatively low. Gene–gene interactions might play a significant role in complex traits or diseases and are one of the many possible factors contributing to the missing heritability. However, to date only a few interactions have been found and validated in GWAS due to the limited power caused by the need for multiple-testing correction for the very large number of tests conducted. Here, we used three types of prior knowledge, known GWAS hits, protein–protein interactions, and pathway information, to guide our search for gene–gene interactions affecting four lipid levels. We identified an interaction between *HMGCR* and a locus near *LIPC* in their effect on high-density lipoprotein cholesterol (HDL-C) and another pair of loci that interact in their effect on low-density lipoprotein cholesterol (LDL-C). We validated the interaction on HDL-C in a number of independent multiple-ethnic populations, while the interaction underlying LDL-C did not validate. The prior knowledge-driven searching approach and a locus-based validation procedure show the potential for dissecting and validating gene–gene interactions in current and future GWAS.

## Introduction

The catalog of genome-wide association studies (GWAS) [1] has collected to date over 1,194 publications since the end of 2008, for a total of over 5,697 single nucleotide polymorphisms (SNPs) that are associated with complex human diseases and other complex traits. However, most these associated SNPs exhibit a small effect size, and collectively only explain a relatively small fraction of additive variance [2], [3], [4], [5]. Specifically, a recent meta-analysis of several GWAS, studying a combined sample size between ∼20,000 to ∼100,000 individuals, identified 95 loci associated with the level of one of total cholesterol (TC), low-density lipoprotein cholesterol (LDL-C), triglyceride (TG), and high-density lipoprotein cholesterol (HDL-C) [6]. In aggregate, these loci explain only 25–30% of heritable variation for each trait [6]. Many hypotheses aiming to explain the missing heritability of GWAS have been proposed, including structural variants, rare variants, gene-environment interactions, epigenetics, and complex inheritance [2], [3], [4], [5]. Because gene-gene (epistatic) interactions may contribute to missing heritability to some extent [7], [8], [9], here we seek to find examples of pairs of loci that interact in their effects on any of the four lipid levels, which are important risk factors of coronary artery disease [10].

Epistasis has been investigated in order to understand the relationship between genotype and phenotype since Bateson [11] discovered in 1905 that some genes can suppress the effects of others. Thereafter, a number of epistatic interactions have been identified in QTL mapping studies or GWAS in humans [12], [13] and other organisms [14], [15], [16]. Studies of model organisms suggest that gene-gene interactions are a common phenomenon [17], [18], [19], [20]. However, they have proven difficult to detect in humans, chiefly due to the limited statistical power associated with the large combinatorial number of tests and the skew towards low minor allele frequencies [18], [21]. Hence, in order to increase power to detect gene-gene interactions in GWAS, a series of methods have been developed to prioritize candidate SNPs using prior knowledge of established GWAS hits [22], and recently also using knowledge of protein-protein interactions (PPIs) [23], [24] and pathway information [25].

Although some interactions affecting complex diseases and traits have been reported in humans [12], [26], replication of these interactions in independent samples has proven difficult [13]. He *et al.* [27] showed that this low replication is in part attributable to low power and small effect sizes of tag SNPs in GWAS. For two interacting causal loci, the observed interaction effect between two respective tag SNPs (each tagging one of the causal loci) is proportional to the underlying causal interaction effect multiplied by the product of the two linkage disequilibrium (LD) coefficients between each tag SNP and the respective causal variant. This decrease in the measured interaction effect reduces the statistical power of the interaction test and it also reduces the probability of replication of significantly identified interactions. This reduction is further exacerbated by heterogeneity in the LD structure between different populations and among population samples. These are the same problems that plague the power of single-marker GWAS tests, but they are exacerbated in interaction testing, with a *quadratic* dependence on LD between markers and causal loci, which lead to a much greater reduction in power. Motivated by this problem, Liu *et al.* [28] proposed a local validation analysis and successfully replicated the loci of a few interactions underlying common human diseases.

In this study, we aim to improve the power to detect gene-gene interactions in existing large-scale GWAS data sets by considering for interaction testing only a highly focused set of candidate SNPs extracted from prior information of known GWAS hits, PPIs, and pathway information. To improve the power of replicating gene-gene interaction signals in independent samples, we introduce an adaptive locus-based validation procedure that follows an approach similar to Liu *et al.* [28]. Applying these procedures for testing for gene-gene interactions underlying lipid levels, we discovered a significant interaction affecting HDL-C levels, which provides new insights into the genetic architecture of this complex trait. Using the adaptive locus-based validation procedure, we also successfully replicated this novel interaction in four independent cohorts, including two cohorts of different ethnicity.

## Results

### Knowledge-driven identification of gene–gene interactions

We tested the statistical significance of gene-gene interaction between each pair of SNPs among 125 SNPs from 95 loci that have been previously individually associated with any of the four lipid levels [6] for a total of 7,750 tests, out of ∼3 trillion possible tests between each pair of SNPs in our data. Tests of interaction were conducted using genotype data or imputed genotypes in a sample of 9,713 European Americans (EAs) from the Atherosclerosis Risk in Communities **(**ARIC) study [29] (Materials and Methods). We used an *F*-test with four degrees of freedom within a linear model framework for interaction testing [30], [31]. This test considers the 3×3 table of genotype pairs for two SNPs and tests for significant interaction between the two SNPs on top of any additive or dominance effects that each of the SNPs might exhibit by itself. For consideration of statistical power and robustness, we discarded from testing pairs of SNPs for which one or more of the 9 genotype-by-genotype combinations appeared in fewer than 20 individuals in our sample (Materials and Methods).

**Figure 1:**
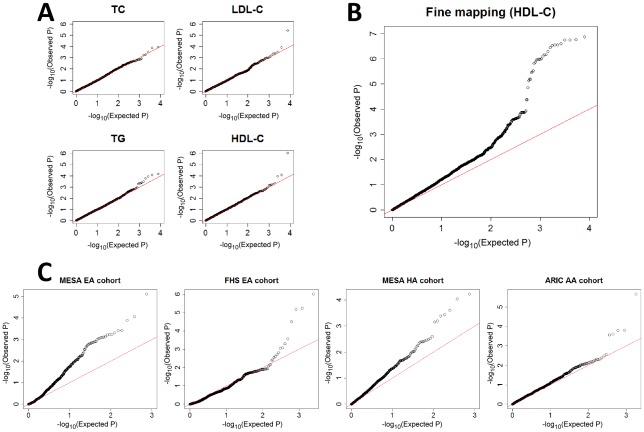
QQ plots for gene–gene interaction tests. A) Discovery in ARIC EA cohort by testing all possible pairs of SNPs among the 125 previously associated SNPs [6], for each of the four traits, showing deviation from expectation for two of them, and pointing in each of these two cases to a single pair of interacting SNPs (Table 1). B) Fine mapping in ARIC EA by testing all possible pairs of SNPs in the 100 kb surrounding rs12916 and rs1532085 that were found from panel A (HDL-C). C) Validation by testing SNP pairs surrounding rs3846662 and rs2043085 (found from panel B; see also Table 1) in four additional cohorts, each pointing to significant gene-gene interaction between the two regions. For all, stage (iii) of the adaptive locus-based validation procedure is shown, though replication has already been successful in stage (ii) in the MESA EA cohort.

Testing for interaction between 7,750 pairs of SNPs for each of four quantitative traits, we identified one significant interaction underlying each of LDL-C level and HDL-C level (Figure 1a). The interaction underlying LDL-C level is between rs2247056 and rs1030431 (Bonferroni corrected *P*_c_ = 0.003; Figure 1a). To explore the interaction between the two loci with better resolution, we tested for interaction between each SNP in the 100 kb surrounding rs2247056 and each SNP in the 100 kb surrounding rs1030431 and found that the interaction signal peaked between rs2853928 and rs1993453 (*P*_c_ = 0.01 after accounting for all additional pairs of SNPs tested; Figure S1). The discovery SNP pairs are in high LD with the fine-mapped SNP pairs, with an *r* ^2^ value of 0.997 between rs2247056 and rs2853928 and 0.999 between rs1030431 and rs1993453. The former two reside near a pseudogene, *LOC100133383*, and the latter two are located near and in gene *UBXN2B*, respectively. However, this suggestive interaction underlying LDL-C did not replicate in independent cohorts.

Henceforth, we focus on the interaction between rs12916 and rs1532085 on HDL-C levels (*P*_c_ = 0.008), since its validation in additional cohorts is highly significant, as described below. We first tested for interaction between each SNP in the 100 kb surrounding rs12916 and each SNP in the 100 kb surrounding rs1532085. While many of these pairs show significant interactions (Figure 1b), as expected from LD, we observed the strongest signal between rs3846662 and rs2043085 (*P*_c_ = 0.002). The fine-mapped pair of SNPs is in high LD with the original pair of SNPs, with an *r* ^2^ value of 0.88 between rs3846662 and rs12916 and an *r* ^2^ value of 0.93 between rs2043085 and rs1532085 (Figure S2). rs3846662 is intronic in *HMGCR* (Table 1), which has not been previously associated with HDL-C, but has been associated with both TC and LDL-C levels [6]. rs2043085 is upstream of *LIPC* (Table 1), which has been previously found to be associated with HDL-C [6].

**Table 1:** Significant interactions on HDL-C in multi-ethnic cohorts.

Test Stage	Cohort^a^	SNP 1				SNP 2				*P_c_* d
		chr	pos^b^	rsID	Gene^c^	chr	pos^b^	rsID	Gene^c^	
Discovery	ARIC EA	5	74656539	rs12916	*HMGCR* (3′ UTR)	15	58683366	rs1532085	40.8 k U *LIPC*	0.008
Fine Mapping	ARIC EA	5	74651084	rs3846662	*HMGCR* (Intron)	15	58680954	rs2043085	43.2 k U *LIPC*	0.002
Validation	MESA EA	5	74651084	rs3846662	*HMGCR* (Intron)	15	58582540	rs1973688	141.6 k U *LIPC*	0.006
Validation	FHS EA	5	74651864	rs55727654	*HMGCR* (Intron)	15	58666341	rs473422	57.8 k U *LIPC*	0.002
Validation	MESA HA	5	74602699	rs1423527	30.3 k U *HMGCR*	15	58718340	rs7163280	5.8 k U *LIPC*	0.04
Validation	ARIC AA	5	74685520	rs3761743	27.6 k D *HMGCR*	15	58736623	rs567838	*LIPC* (Intron)	0.004

a EA denotes European American; HA denotes Hispanic American; AA denotes African American.

b Build 37.1 (GRCh37).

c U indicates upstream of; D indicates downstream of.

d *P*-value after Bonferroni correction.

**Figure 2:**
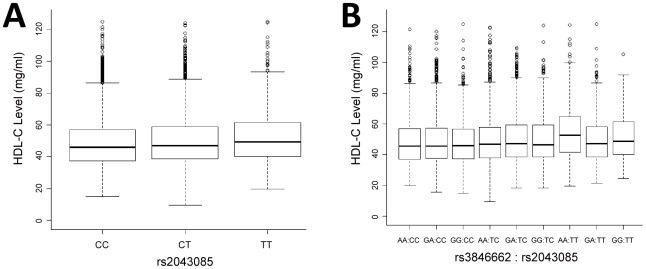
Marginal and interaction effect sizes on HDL-C level in ARIC EA cohort. A) Boxplot of the effect sizes of rs2043085. Allele T of rs2043085 leads to increased HDL-C, with median levels of 45.8, 46.9 and 49.3 mg/ml for CC, CT, and TT (difference in mean levels reported in main text). B) Boxplot of the effect sizes of the SNP pair, rs3846662∶rs2043085. The genotype AA∶TT leads to a considerable increase of HDL-C. The median HDL-C level in the entire sample is 46.7 mg/ml, while the median level for AA∶TT is 52.7 mg/ml (rs2043085 is the only one of the two that is associated by itself with HDL-C, hence shown in panel A).

The interaction between rs3846662 and rs2043085 affects HDL-C twice as much as the effect of the polymorphism in *LIPC* alone: While individuals with TT genotype at rs2043085 already exhibit an average increase of 2.63 mg/ml in HDL-C (standard error (SE) = 0.014; Figure 2a), this genotype in combination with an AA genotype at rs3846662 leads to an average increase of 5.72 mg/ml (SE = 0.041; Figure 2b). The linear model with these two SNPs has an R-square value of 0.5% and the linear model with the two SNPs and their interaction has an R-square value of 0.8%, which indicates that the interaction explains additional 0.3% of the overall variation in HDL-C levels (Materials and Methods; Table S1). We tested whether rs3846662 and rs2043085 exhibit gene-gene interactions underlying any of the other lipid levels, and found a nominally significant interaction underlying LDL-C (*P* = 0.028), and almost significant interaction underlying TG (*P* = 0.08) in ARIC.

We performed a larger scale interaction analysis between all pairs of SNPs that (i) are found in interacting genes according to a curated human protein-protein interaction network (∼6 million pairs), or (ii) are involved in the pathway of metabolism of lipids and lipoproteins (∼27 million pairs). All SNPs in a gene were considered, as well as in the 5 kb regions upstream and downstream. This analysis detected no significant gene-gene interactions following Bonferroni correction (*P*_c_≥0.58 for PPIs; Figure S3; *P*_c_≥0.14 for pathway; Figure S4).

### Validation of gene–gene interaction in pairs of loci

Considering the quadratic reduction in replication power as a function of LD between tag SNPs and causal loci, we aimed to increase power via an adaptive locus-based validation procedure that is related to that of Liu *et al.* [28]. In considering a replication dataset, the procedure follows three sequential stages that leverage the signals of proxy markers: (i) test for interaction between the original SNP pair between which gene-gene interaction has been detected; (ii) test for interactions between each of the two original SNPs and each SNP in the proximate region containing the other original SNP; (iii) test for interactions between each pair of SNPs in each of the two respective proximate regions containing the two original SNPs. This validation procedure proceeds sequentially and stops at any stage when significant interactions were detected after multiple-testing correction. Both the method of Liu *et al.* and our adaptive locus-based validation method focus on replicating the interaction between a pair of loci, rather than between a pair of SNPs, due to the power limitations of replicating an interaction between SNPs. The null hypothesis of the entire three-stage procedure is that there is no interaction between the pair of loci, rather than just between the pair of SNPs, thus the procedure continues sequentially as described to consider proxy SNPs from the loci containing each original SNP. Replication is successful if an interaction between any SNP pair from the two loci is significant after multiple-testing correction. Similar locus-based approach has also been used in the context of gene-based GWAS tests for single-marker association, which use an entire gene or locus as the testing unit of association, rather than a single SNP [27], [32].

To validate the gene-gene interaction affecting HDL-C, we performed replication analyses in two additional GWAS datasets from the Framingham Heart Study (FHS) [33] and the Multi-Ethnic Study of Atherosclerosis (MESA) [34], as well as in the African American (AA) cohort from the ARIC study [29]. Using our adaptive locus-based procedure, we tested for interaction sequentially between SNPs surrounding rs3846662 and SNPs surrounding rs2043085. We observed significant interactions in the two additional EA cohorts from FHS and MESA (Figure 1c), with *P*_c_ = 0.002 and *P*_c_ = 0.006 for the most significantly interacting SNP pair (Table 1). Replication was also significant in Hispanic Americans (HA) from MESA and AAs from ARIC (Figure 1c; Table 1). The R-square of linear model with the two interacting SNPs varies between 0.2–0.5% across the four replication cohorts, with the interaction term between the two explaining an additional 0.2–1.1% of the overall variation in HDL-C levels (Table S1). The replication procedure failed in a sample of AAs from MESA (Figure S5).

None of the successful replications were replicated at stage (i) of the adaptive locus-based validation procedure, which means that an interaction between the same SNP pair is not observed significantly in the additional samples. The interaction was successfully validated in stage (ii) of the three stages in the MESA EAs, with the same SNP in *HMGCR* (rs3846662) and a proxy SNP near *LIPC* exhibiting a significant gene-gene interaction after multiple-testing correction. The other three successful replications occurred at stage (iii) (Table 1), emphasizing the importance of a locus-based replication approach. The combined evidence from the discovery and four different validation cohorts for a gene-gene interaction between the two loci under study is overwhelmingly significant, even following a conservative Bonferroni correction (*P*_c_ = 9.0×10^−8^).

While the gene-gene interaction signal peaks for different pairs of SNPs across the different cohorts (Table 1), the type of interaction and effect patterns appear consistent across several sample sets (Figure S6). To test this formally, we partitioned the significant SNP-SNP interactions into the four possible interaction components on top of the marginal SNP effects, namely additive by additive (A×A), additive by dominance (A×D), dominance by additive (D×A), and dominance by dominance (D×D) components (Materials and Methods). Considering a nominal significance level of 0.01, D×A and D×D components are significant and underlie the significant interaction in the ARIC EA discovery set, between rs12916 and rs1532085 (Table S1). All four terms are significant between the pair of SNPs, rs3846662 and rs2043085, that resulted from fine mapping in the same discovery set, with D×A and D×D being of the same effect direction (sign) and similar effect sizes as between rs12916 and rs1532085 (Table S1). Examining the two replication cohorts of a similar (EA) ancestry, the interaction in the MESA cohort similarly shows significant D×A and D×D components, with same effect direction, though with larger effect sizes and a higher proportion of phenotypic variance explained (Table S1). None of the four terms is significant by itself in the EA FHS cohort. These results of consistent patterns of interaction across the EA cohorts support the possibility that they are all governed by the same (unobserved or partially unobserved) interacting variants.

### Validation of imputation accuracy

To verify that our results are not an artifact of imputation errors, we compared imputed genotypes of the two SNPs (rs12916 and rs3846662) that were involved in significant interactions and for which we could obtain measured genotype data from an independent source, using the ITMAT/Broad/CARE (IBC) Vascular Disease 50 k SNP Array chip [35]. For these two SNPs, *r* ^2^ between imputed and actual genotypes is 0.914 and 0.921 and the genotype concordance rate is 94.5% and 94.7%, respectively. Although the imputation is not perfect, the two interaction tests involving these two SNPs are at least as significant when replacing imputed genotypes with measured IBC genotypes, consistent with imputation errors adding noise and masking some of the signal, rather than biasing the statistical test.

## Discussion

Tests of gene-gene interactions are not as powerful as tests of single-marker association, so a judicious strategy is essential for successful interaction analysis in GWAS [9], [36]. The first step is to determine the size of the analysis, genome-wide or focusing on candidate SNPs. This step should consider the sample size, possible effect size of the underlying interaction, and the desired statistical power. Current single-marker GWAS have been successful in detection of single-marker associations for many complex diseases or traits using a stringent genome-wide significance level (*P*<5×10^−8^). To achieve a similar success for interaction analysis, we are limited to performing ∼1 million tests even if the interaction test and single-marker test had the same statistical power. This limitation means that we are not able to conduct an inclusive all-by-all pair-wise interaction analysis in current GWAS. Thus, in this study we only tested for interactions between candidate SNPs based on prior knowledge.

We used three types of prior knowledge, known GWAS hits, protein-protein interaction networks, and known functional pathways. These three analyses might be different in the enrichment of epistasis signals and are also different in the number of interaction tests, 7,750 based on known GWAS hits, ∼6.2 million using PPI, and ∼27 million with pathway information. We found significant interactions from the 7,750 interaction tests using known GWAS hits. As the sample size of ∼10,000 individuals is relatively large among existing GWAS, this indicates that the observed (tagged) effect size of any other underlying interactions is no larger than the marginal effects of single SNPs. It is also likely that the epistasis signals are better enriched between markers that are marginally associated with lipid traits such that testing interactions among known GWAS hits is more powerful in our study. Therefore, our results suggest that a small-scale interaction analysis of candidate SNPs driven by known marginal associations might be a good choice for detecting epistatic interactions in current GWAS.

Recently, the Population Architecture using Genomics and Epidemiology Study [37] found only ∼50% of the 125 reported associations with lipid levels [6] to replicate in three non-European cohorts. Due to the quadratic decrease in the interaction effect of tagged markers, gene-gene interactions are even less likely to replicate in diverse populations. Leveraging signals from proximate linked SNPs, our adaptive locus-based method successfully validated gene-gene interactions between *HMGCR* and *LIPC* in four additional, independent cohorts, including two of non-European ancestry. Although the most significant interaction in each cohort involves different SNPs, they are proximate across the cohorts, with stronger LD and smaller distances amongst the three EA cohorts and weaker LD and larger distances between them and the HA and AA cohorts (Figure S2 and Table 1). The differences in distance and LD between ethnicities could be due to differences in genetic background, demographic history, and natural selection, even if the different SNP pairs capture the same underlying causal interaction. However, the interaction shows similar patterns among some, but not all cohorts (Figure S6 and Table S1), while the different SNPs around *HMGCR* are in strong LD, and those around *LIPC* show weak LD (Figure S2). These results suggest that the five SNP pairs either capture separate causal interactions or are only in weak LD with the same pair of interacting, unobserved variants.

Another possibility is that the interaction is between relatively rare causal variants: Much like rare causal variants can lead to multiple independent associations of common variants, dubbed “synthetic associations” [38], an interaction between two rare causal variants can produce an even larger number of independent “synthetic interactions”, which can in principle explain almost-independent, yet proximate gene-gene interactions. Another possibility is that the underlying interaction is more complex and involves more than a pair of SNPs. In that case, in our analysis of pairs of SNPs, each pair might tag only certain aspects of the underlying interaction.

Both *HMGCR* and *LIPC* are involved in metabolism of lipids and lipoproteins. *HMGCR*, which has been associated with TC and LDL-C [6], regulates the rate of cholesterol synthesis via a negative feedback mechanism mediated by sterols and non-sterol metabolites [39]. *LIPC* encodes hepatic lipase which is an important enzyme in HDL metabolism [40] and has been previously associated with HDL-C levels [6]. The interaction between variants in these genes as discovered in this study can be possibly explained by an indirect interaction between cholesterol synthesis and the metabolism of LDL and HDL particles. *HGMCR* is the rate-controlling enzyme in the mevalonate pathway for cholesterol synthesis [41]. Much of this cholesterol will form cholesteryl esters that will be packaged into various lipoproteins including LDL, HDL, and TG-rich lipoproteins. There are a number of known lipoprotein interactions that result in the flow of cholesterol in the form of cholesteryl esters from LDL and VLDL to HDL-C [42]. This cholesterol is later processed with the HDL particle by either reabsorbing into the liver or excretion in the urine [43].

The rs2043085 SNP in the *LIPC* gene region, where our strongest signal has been observed in fine mapping in the discovery panel, was recently associated with elevated HDL-C in an additional cohort of individuals with mixed dyslipidemia [44]. Increased HDL-C may be related to modest inhibition of TG hydrolysis in the HDL particle by hepatic lipase, slowing its excretion in the urine along with its cholesterol content. Because *HMGCR* has a major effect on cholesterol synthesis, it will also indirectly affect the cholesterol content in the HDL particle through its interaction with LDL and TG-rich particles. In addition, *LIPC* has been reported to exhibit gene-gene interaction with other genes associated with lipid traits [45], [46], and *HMGCR* has been reported to interact with *ABCA1* in Alzheimer's disease risk [47]. While these results increase the plausibility of a biological interaction between these two genes, we note that a statistical gene-gene interaction does not necessarily entail an underlying epistatic interaction in the biological sense [7]. We also note that while we refer to the interaction as being between *HMGCR* and *LIPC*, these two genes are implicated only by genomic proximity, and we presented no direct evidence that these genes are the interacting functional units.

We conclude that a focused study with higher enrichment of putative signals might have improved power to detect gene-gene interactions underlying complex diseases or traits. By focusing only on SNPs that were previously associated with the studied trait, HDL-C level, or any of a handful of related traits (other lipid levels), we successfully identified an interaction between SNPs in or near *HMGCR* and SNPs upstream of *LIPC* in European American samples. By using a locus-wide validation procedure to overcome the quadratic impact of partial SNP tagging on the observed interaction effect size, we further replicated the interaction between these loci in additional European American samples, as well as in African American and Hispanic American samples.

## Materials and Methods

### Study descriptions

All work done in this paper was approved by local institutional review boards or equivalent committees.

#### Atherosclerosis Risk in Communities (ARIC) Study

The ARIC Study is a multi-center prospective investigation of atherosclerotic disease [29]. EA and AA individuals aged 45–64 years at baseline were recruited from four communities: Forsyth County, North Carolina; Jackson, Mississippi; suburban areas of Minneapolis, Minnesota; and Washington County, Maryland. A total of 15,792 individuals participated in the baseline examination in 1987–1989, with three triennial follow-up examinations. We conducted a discovery interaction analysis using 9,713 EAs from this study, for whom phenotype and genotype data were available, and considered 3,207 AAs from this study as one of the replication cohorts.

#### Framingham Heart Study (FHS)

The FHS is a three generational prospective cohort [33]. 5,209 EAs were initially recruited in 1948 in Framingham, Massachusetts to evaluate cardiovascular disease risk factors. The second generation cohort (5,124 offspring of the original cohort) was recruited between 1971 and 1975, and lipid measurements were obtained multiple times. The third generation cohort (4,095 grandchildren of the original cohort) was collected between 2002 and 2005, and one lipid measurement was obtained. We considered as one of the replication cohorts a sample of 6,575 individuals from FHS for whom genotypes and lipid measurements were available, while accounting for their relatedness (see *Population stratification and relatedness*).

#### Multi-Ethnic Study of Atherosclerosis (MESA)

MESA is a prospective cohort study of 8,296 men and women aged 45–84 years recruited from 6 US communities (Baltimore, MD; Chicago, IL; Forsyth County, NC; Los Angeles County, CA; northern Manhattan, NY; and St. Paul, MN) [34]. MESA was designed to determine the characteristics of subclinical cardiovascular disease and its progression, hence adults were considered and individuals with symptoms or history of medical or surgical treatment for cardiovascular disease were excluded. Participants were enrolled between July 2000 and August 2002 and self-reported their race/ethnicity group as Caucasian or white, African American or black, Spanish/Hispanic/Latino, or Chinese American. We attempted replication in three cohorts from the first three of these ethnicities, with 2,685, 2,588, and 2,174 individuals, respectively, for which genotypes and lipid measurements were available. We discarded 777 Chinese Americans from our replication analysis because of the small sample size.

### Genotype data

We obtained Affymetrix 6.0 SNP array genotyping of samples from the ARIC study [29]. We obtained Affymetrix 6.0 SNP array genotyping of MESA samples and Affymetrix 500 K SNP array genotyping of FHS samples from the database of Genotypes and Phenotypes (dbGaP; *MESA SHARe*, downloaded in May 2011 and *Framingham Cohort*, downloaded in April 2010) [48], [49]. Genotype quality control (QC) steps included the exclusion of individuals with >10% missing data, and the exclusion of SNPs with call rates <90%, minor allele frequencies (MAF)≤1%, or Hardy-Weinberg Equilibrium (HWE) test with *P*<10^−6^. For the pairwise interaction test of each pair of SNPs we also required (i) sample size of each of the nine possible genotype-by-genotype combinations of the two SNPs being >20 in the discovery analysis and >10 in the validation analysis; and (ii) LD of *r* ^2^<0.1 between the two SNPs between which interaction is tested. The first requirement is a generalization of the MAF requirement in single-marker analysis.

We used IMPUTE2 [50] with HapMap3 [51] and 1000 Genomes [52] reference haplotypes to impute untyped SNPs, resulting in the same set of SNPs across cohorts. We did not impute untyped SNPs in MESA HA samples since no appropriate reference panel was available at the time we conducted our analysis. We discarded imputed SNPs with information score less than 0.6. Following this QC stage, we considered the genotype with the maximum posterior probability, and discarded SNPs for which this probability is <0.8.

### Lipid level measurements

We considered four lipid measurements: TC, LDL-C, TG, and HDL-C. All measurements were done in the fasting state using standard enzymatic methods. In all three studies, each lipid level is measured at multiple time points and we considered the average level per individual of each lipid in all our analyses. We applied a log transformation to TG levels to normalize them in face of the skewness in the original distribution, as previously proposed [6]. We excluded individuals known to be taking lipid-lowering medications.

Gender, age, age squared, and body mass index (BMI) were included as covariates in all analyses, similarly to GWAS based on these phenotypes [6], [26]. We averaged values for age and BMI whenever multiple measurements were available, in line with the averaging of lipid levels [6]. The average age was also squared and included as a covariate. Plate is also included as a covariate in the ARIC data since it is correlated with some of the lipid levels (“plate effect”; data not shown).

### Population stratification and relatedness

Principal component (PC) analysis was conducted using EIGENSOFT [53]. Top 10 PCs were included in the analysis as covariates to account for potential population stratification in each of the ARIC and MESA cohorts. For FHS, we applied a mixed model method to account for relatedness by performing the interaction test on the residuals after removing familial structure [26], [54].

### Gene–gene interaction test

As described in [30], [31], we tested for interaction between two SNPs on a quantitative trait as follows. Assume *Y* is the trait of interest and *G_i_* is the genotype of SNP *i* (*i* = 1, 2). *G_i_* denotes the number of copies of the reference allele (0, 1, or 2). Two indicator variables *x_i_* and *z_i_* are defined for each SNP as
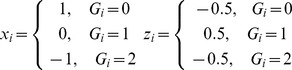
Two linear models were fitted. The first, model (1), allows for additive and dominance effects at each SNP, but is strictly additive (i.e. no interaction) over the two SNPs. The second, model (2), allows for the four possible forms of genotype-by-genotype interaction (additive×additive, additive×dominance, dominance×additive, and dominance×dominance) [55], as follows:
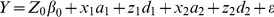
(1)

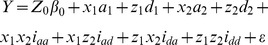
(2)Here, *β*_0_ denotes a vector of intercept and covariates as described above. *a_i_* and *d_i_* denote the additive and dominance effects of SNP *i*, and *i_aa_*, *i_ad_*, *i_da_*, and *i_dd_* are the four interaction effects between the two SNPs.

We tested for the existence of an epistatic interaction of any type by an *F*-test with four degrees of freedom between models (1) and (2) [18]. The *F*-test with four degrees of freedom tends to be more powerful when little is known about the underlying epistatic effect in terms of the possible directions of the deviation from independence of the additive effects. This test is similar to the “–epistasis” option in PLINK [56], except that only additive effects and their interaction are considered in PLINK, and an *F*-test with one degree of freedom is hence applied. We also considered a test for “physiological epistasis” [7] under the same model and obtained very similar results (data not shown). Throughout the results, we report *P* values following a conservative Bonferroni correction. To compare the effects of the different SNP pairs detected in our discovery and validation analyses, we also estimated and tested the four interaction terms in model (2) for each pair of SNPs from different cohorts using a t-test.

### Prior knowledge driven searching strategy

Although we only focus on pairwise interaction analysis, the total number of potential pairwise interaction tests across 2.5 million SNPs is still huge, about 3 trillion tests. Due to the huge reduction in power entailed by multiple-testing correction for such a large number of tests, it is crucial to restrict the number of tests a priori. We aimed to enrich possible interaction signals in the limited number of tests we considered through the following three strategies.

#### GWAS hits

In total 95 loci were recently associated with TC, HDL-C, LDL-C, or TG in a GWAS meta-analysis [6]. We exhaustively tested the pairwise interactions among all the significantly (*P*<5×10^−8^) associated SNPs in these 95 loci, for a total of 125 significant SNPs. For this approach, the total number of interaction tests is 7,750 for each trait.

#### PPI

We assembled over 3000 high-confidence human PPIs and for each exhaustively tested the pairwise interactions between each SNP in the first gene and each SNP in the second gene. For *n*_1_ and *n*_2_ being the numbers of SNPs in the first and second gene, respectively, the number of interaction tests is *n*_1_×*n*_2_ for this PPI. Repeating this process for the 3000 PPIs, we tested a total of ∼6.2 million SNP-SNP interactions. We obtained gene information (hg18) from UCSC genome browser (http://genome.ucsc.edu/) to map SNPs to genes, considering for each gene all SNPs from 5 kb upstream to 5 kb downstream of the gene. These PPIs, however, have no specific implications to lipid levels as they are not context-based, and were collected under different physiological conditions.

#### Functional pathways

We tested for gene enrichment of the 96 genes reported in ref. 6 as associated with lipid levels. As expected, the metabolism of lipids and lipoproteins pathway (www.reactome.org) is the most significant pathway (*P*<10^−20^). There are a total of 228 genes in this pathway, to which we mapped a total of 12,716 SNPs similarly to above. We tested for pairwise interactions between each pair of these 12,716 SNPs, yielding a total of ∼27 million tests.

### Adaptive locus-based validation method

Liu *et al.* [28] developed a local validation strategy and validated a few interactions affecting common human diseases. This strategy attempts to replicate the interaction between two loci rather than the interaction between the original pair of SNPs. To further improve power, we extended this local validation strategy to an adaptive locus-based validation procedure: For a detected interaction between SNP A and SNP B in the discovery panel we followed three stages in each of the validation panels. (i) First, test for interaction between SNP A and SNP B; (ii) Second, if the interaction in (i) is not significant by itself, test for interaction between A and each SNP<200 kb away from B, and similarly between B and each SNP surrounding A; (iii) Last, if no test in the second stage is significant following multiple-hypothesis correction, test for interaction between each SNP<100 kb away from A and each SNP<100 kb away from B. Assuming *n*_1_ and *n*_2_ SNPs in the locus surrounding A and B, respectively, the number of interaction tests performed is 1, *n*_1_+*n*_2_, and *n*_1_×*n*_2_ in the three stages, respectively, with *n*_1_ and *n*_2_ in stage (iii) being smaller than those in stage (ii) due to considering only 100 kb. To maintain power in light of multiple-testing correction, the validation process proceeds sequentially and stops once we find significant results after multiple-testing correction. The interaction between rs3846662 and rs2043085 on HDL-C was successfully validated in stage (ii) for MESA EA samples and in stage (iii) for the MESA HA, FHS EA, ARIC AA cohorts. It did not validate significantly after multiple-testing correction in any of the three stages in the MESA AA samples. We used the same procedure as in step (iii) for fine mapping within the discovery panel.

## Supporting Information

Figure S1 Quantile–quantile (QQ) plots for gene–gene interaction tests of LDL-C in ARIC EA cohort. (A) Discovery analysis (reproduced from Figure 1a in main text); (B) Fine mapping by testing all possible pairs of SNPs in the 100 kb surrounding each of rs2853928 and rs1993453.
(TIF)

Figure S2 Linkage disequilibrium in data from the 1000 Genomes Project of all SNPs involving in significant interactions underlying HDL-C in any of the cohorts (i.e. all SNPs from Table 1). (A) and (C) are for SNPs in the locus on chromosome 5 in the CEU (European American) and YRI (West African) 1000 Genomes samples, respectively; similarly, (B) and (D) for SNPs on the interacting locus on chromosome 15. These figures were produced by Haploview [57]. The numbers shown are R-square values with zeroes and dots omitted.
(TIF)

Figure S3 QQ plots for gene–gene interaction tests in ARIC EA cohort based on the PPI-based strategy for considering pairs of SNPs. (A) TC; (B) LDL-C; (C) TG; (D) HDL-C.
(TIF)

Figure S4 QQ plots for gene–gene interaction tests in ARIC EA cohort based on the pathway-based strategy for considering pairs of SNPs. (A) TC; (B) LDL-C; (C) TG; (D) HDL-C. We found a deviation in the QQ plot of the *P* values for interactions underlying TC levels and the strongest interaction signal appears between rs4804546 and rs914196, though it is not significant following correction for the ∼27 million tests (*P*_c_ = 0.14). The two genes from the pathway of metabolism of lipids and lipoproteins associated with this interaction are *CARM1* and *AGPAT3*. *AGPAT3* was previously found to be associated with the level of phospholipid [58], while *CARM1* has not been associated to the best of our knowledge with any lipid levels.
(TIF)

Figure S5 QQ plots for stage (iii) of the adaptive locus-based validation tests in MESA African American cohort, which show no significant results.
(TIF)

Figure S6 Effect sizes on HDL-C level of the six SNP pairs from Table 1 in main text in the respective cohorts. The ARIC EA fine mapping panel is reproduced from Figure 2B in main text.
(TIF)

Table S1 Effect estimates for significant interactions between SNPs surrounding *HMGCR* and *LIPC* on HDL-C in EA, AA, and HA cohorts.
(DOC)
