# Supplementary figures and images for: Knowledge-Driven Analysis Identifies a Gene–Gene Interaction Affecting High-Density Lipoprotein Cholesterol Levels in Multi-Ethnic Populations
Source: PLoS Genet. 2012 May 24;8(5):e1002714. doi: 10.1371/journal.pgen.1002714 (PMC3359971; doi:10.1371/journal.pgen.1002714)

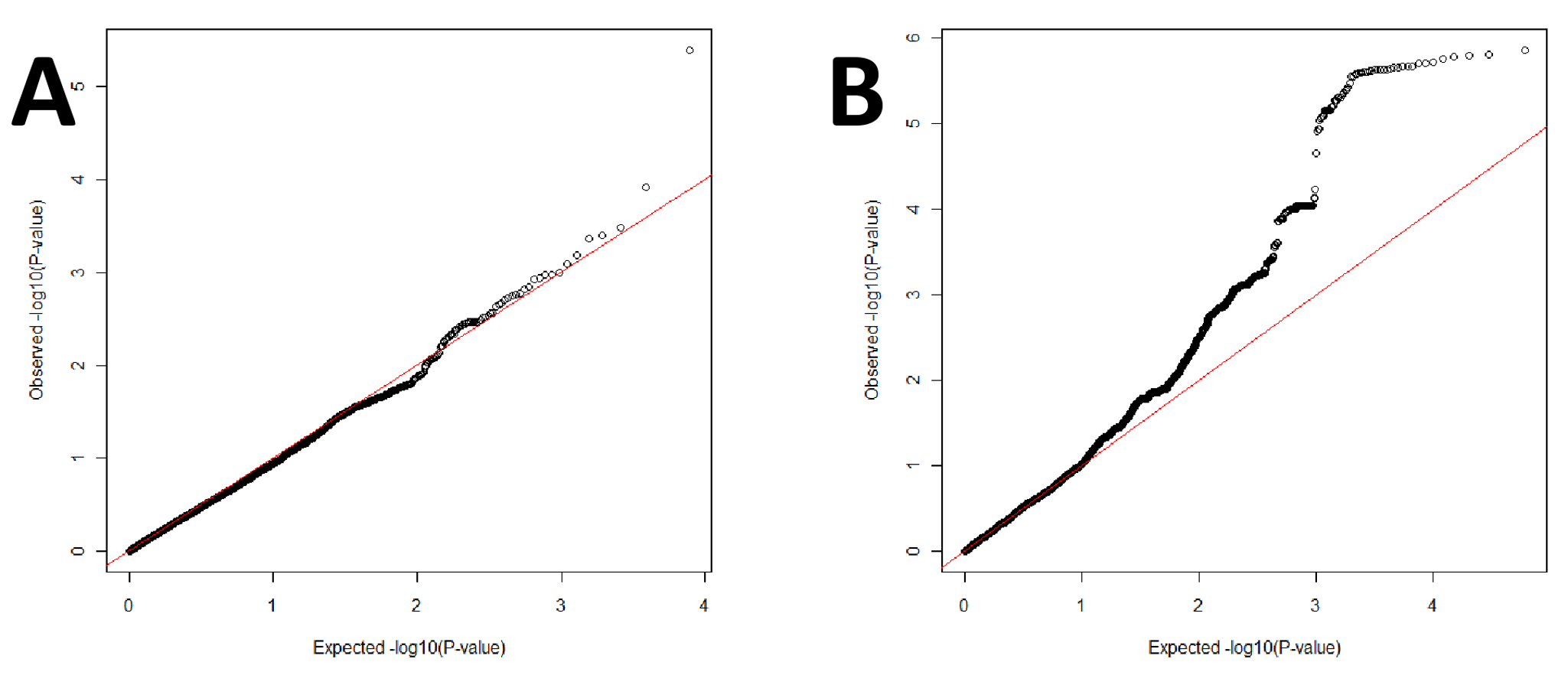

Supplement: Figure S1 — Quantile–quantile (QQ) plots for gene–gene interaction tests of LDL-C in ARIC EA cohort. (A) Discovery analysis (reproduced from Figure 1a in main text); (B) Fine mapping by testing all possible pairs of SNPs in the 100 kb surrounding each of rs2853928 and rs1993453. (TIF) [file pgen.1002714.s001.tif]

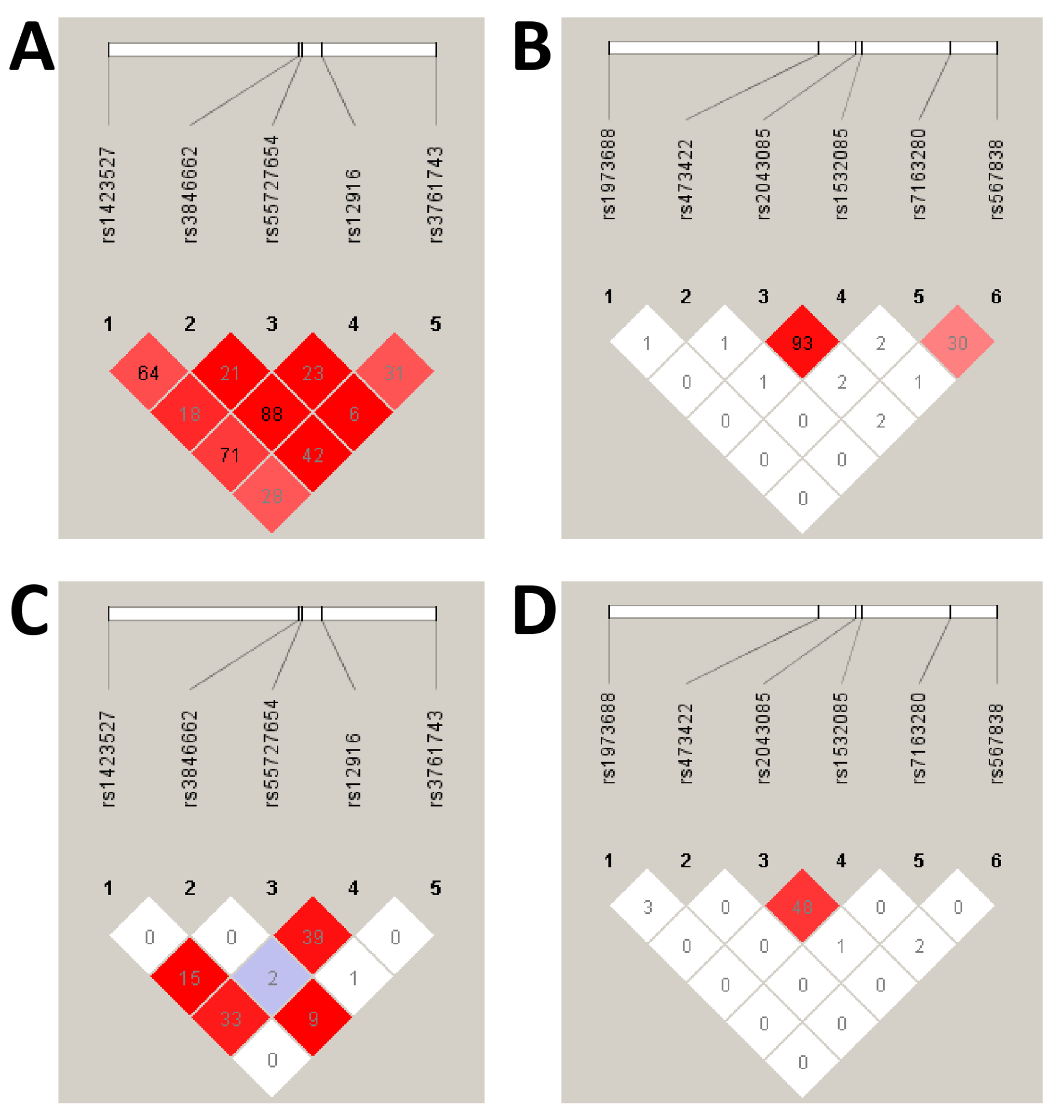

Supplement: Figure S2 — Linkage disequilibrium in data from the 1000 Genomes Project of all SNPs involving in significant interactions underlying HDL-C in any of the cohorts (i.e. all SNPs from Table 1). (A) and (C) are for SNPs in the locus on chromosome 5 in the CEU (European American) and YRI (West African) 1000 Genomes samples, respectively; similarly, (B) and (D) for SNPs on the interacting locus on chromosome 15. These figures were produced by Haploview [57]. The numbers shown are R-square values with zeroes and dots omitted. (TIF) [file pgen.1002714.s002.tif]

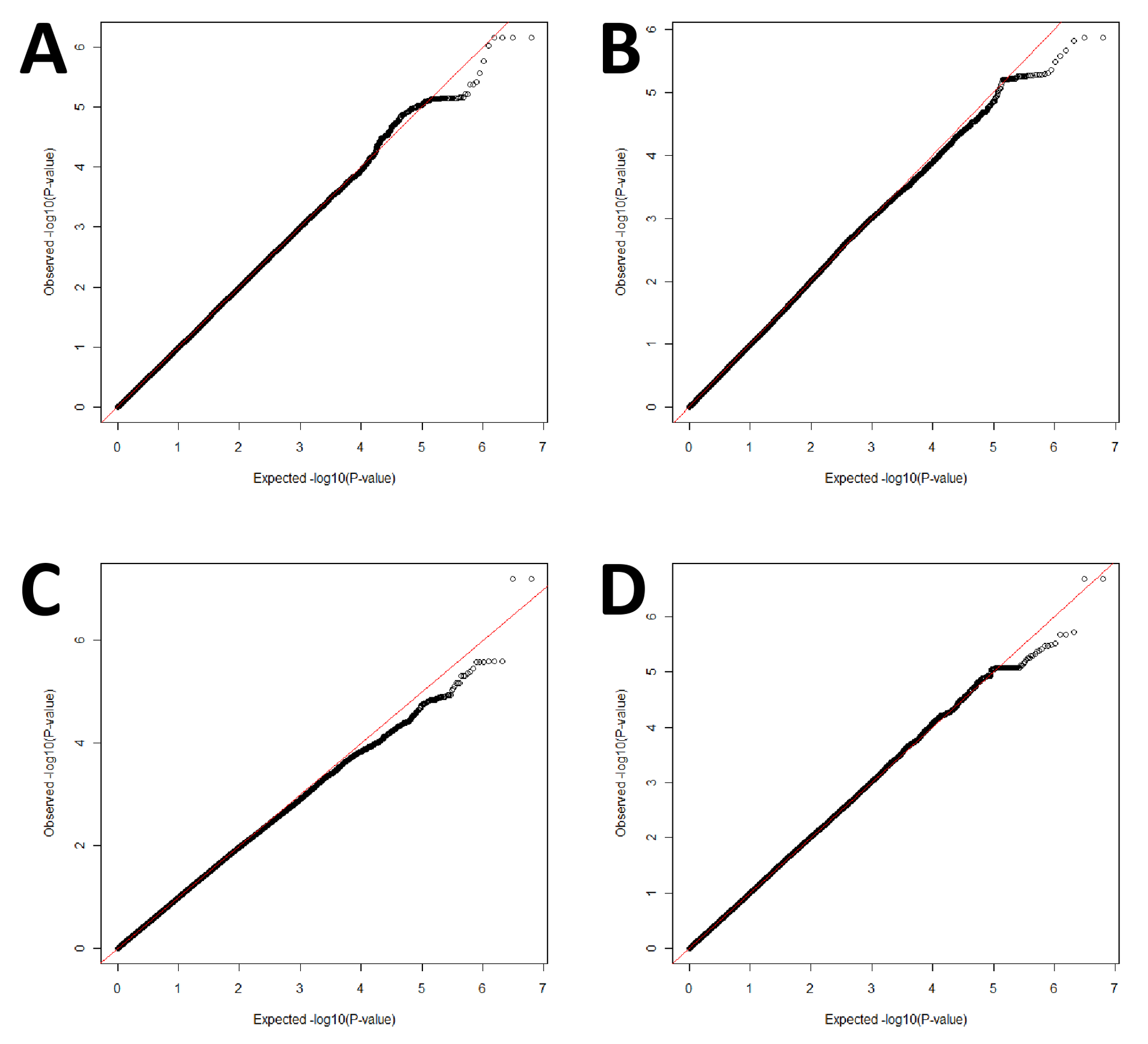

Supplement: Figure S3 — QQ plots for gene–gene interaction tests in ARIC EA cohort based on the PPI-based strategy for considering pairs of SNPs. (A) TC; (B) LDL-C; (C) TG; (D) HDL-C. (TIF) [file pgen.1002714.s003.tif]

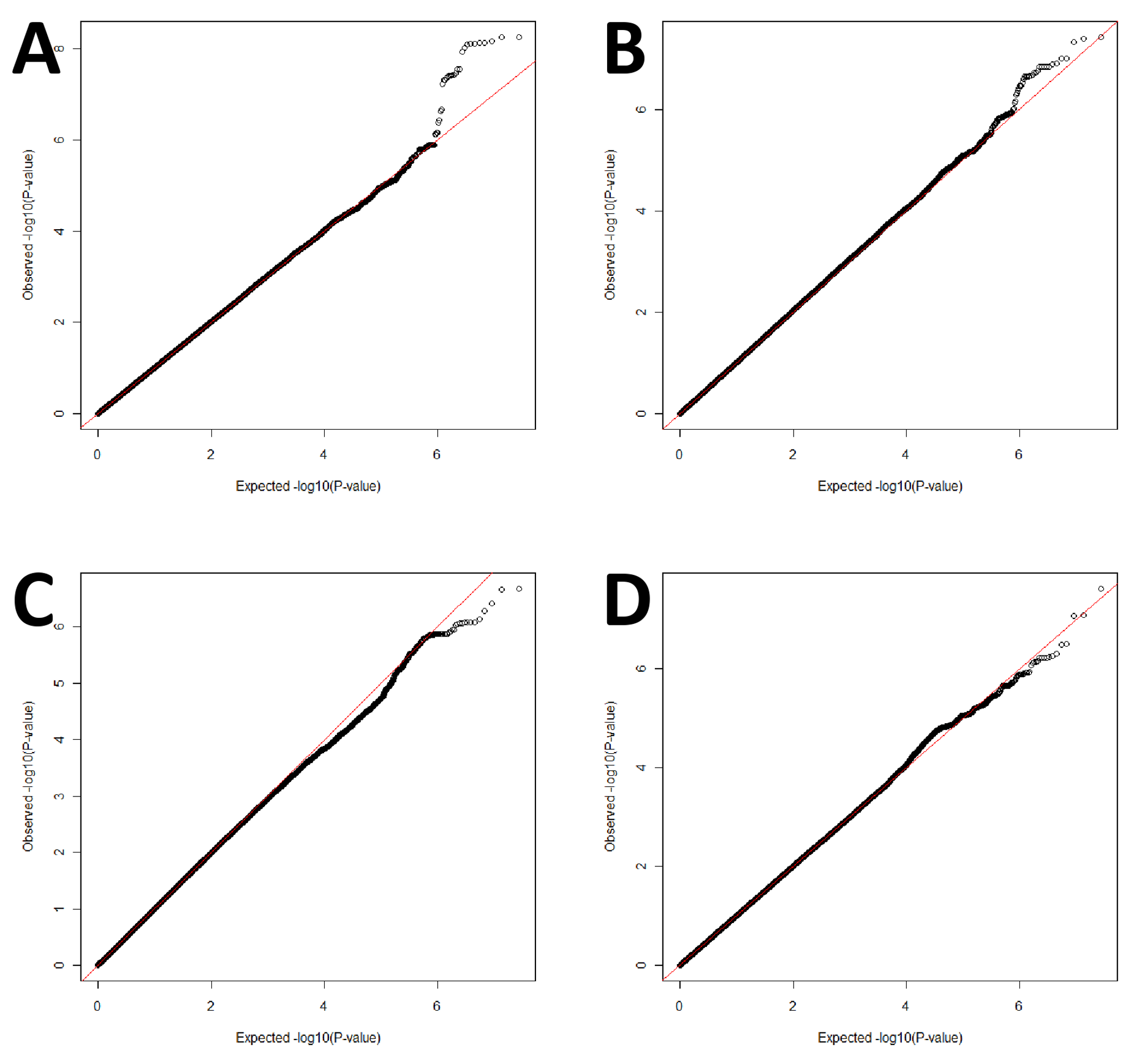

Supplement: Figure S4 — QQ plots for gene–gene interaction tests in ARIC EA cohort based on the pathway-based strategy for considering pairs of SNPs. (A) TC; (B) LDL-C; (C) TG; (D) HDL-C. We found a deviation in the QQ plot of the P values for interactions underlying TC levels and the strongest interaction signal appears between rs4804546 and rs914196, though it is not significant following correction for the ∼27 million tests (P c = 0.14). The two genes from the pathway of metabolism of lipids and lipoproteins associated with this interaction are CARM1 and AGPAT3. AGPAT3 was previously found to be associated with the level of phospholipid [58], while CARM1 has not been associated to the best of our knowledge with any lipid levels. (TIF) [file pgen.1002714.s004.tif]

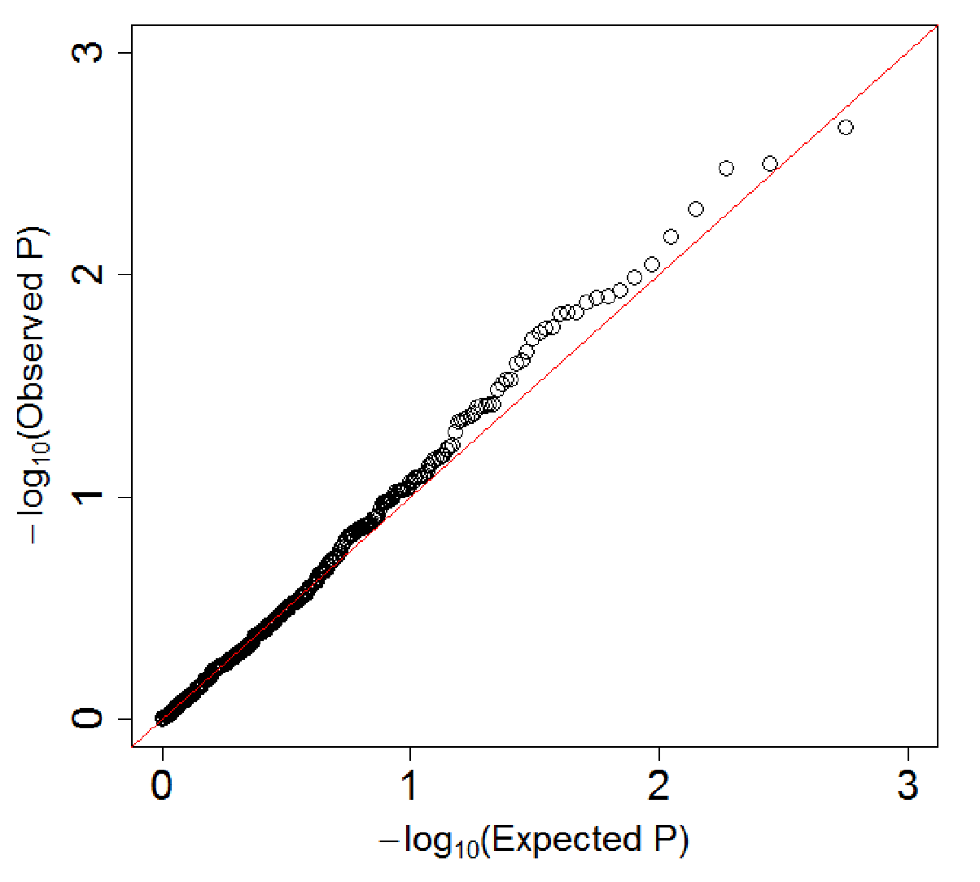

Supplement: Figure S5 — QQ plots for stage (iii) of the adaptive locus-based validation tests in MESA African American cohort, which show no significant results. (TIF) [file pgen.1002714.s005.tif]

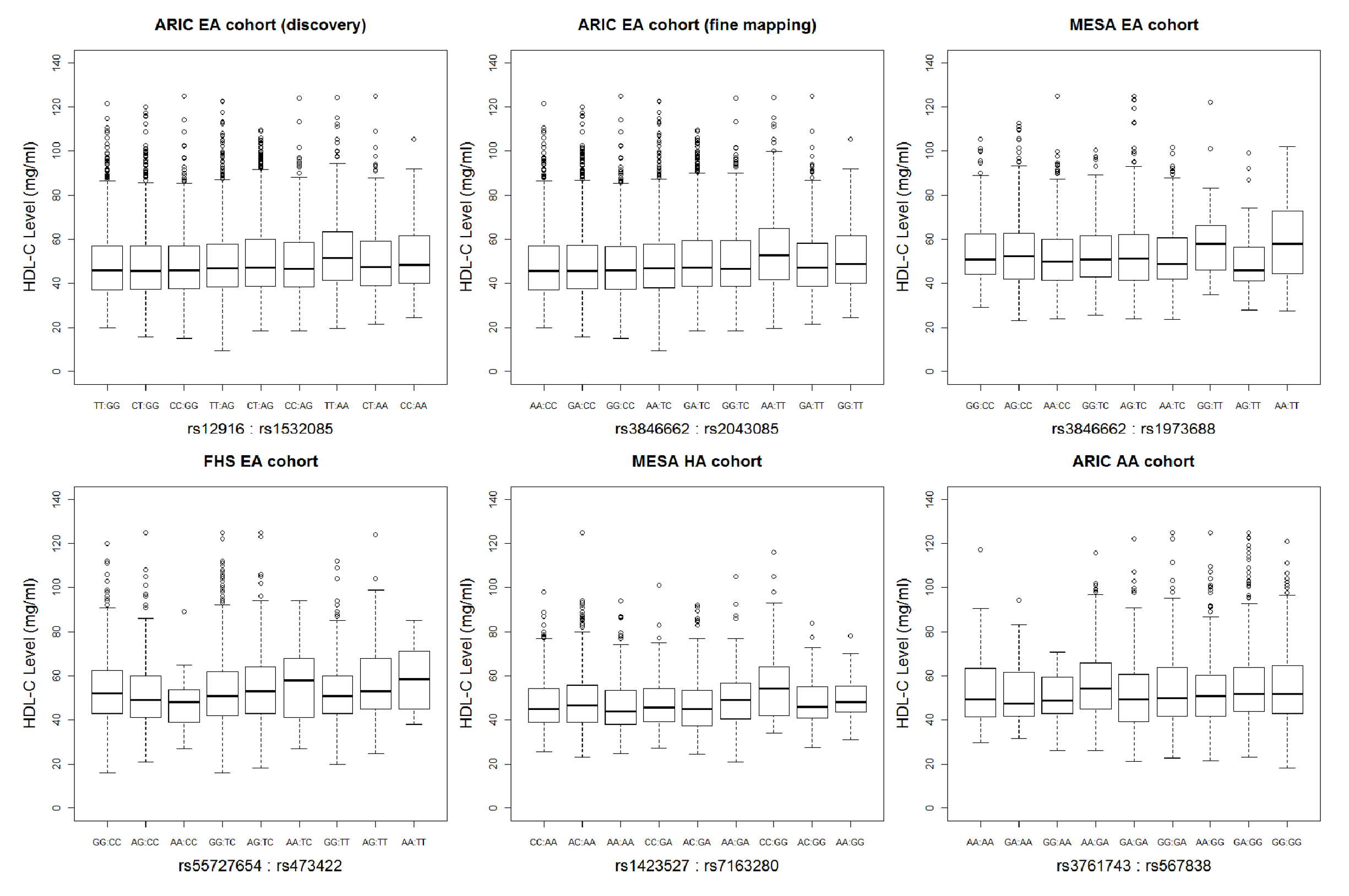

Supplement: Figure S6 — Effect sizes on HDL-C level of the six SNP pairs from Table 1 in main text in the respective cohorts. The ARIC EA fine mapping panel is reproduced from Figure 2B in main text. (TIF) [file pgen.1002714.s006.tif]
