# Supplementary material for: Knowledge-Driven Analysis Identifies a Gene–Gene Interaction Affecting High-Density Lipoprotein Cholesterol Levels in Multi-Ethnic Populations
Source: PLoS Genet. 2012 May 24;8(5):e1002714. doi: 10.1371/journal.pgen.1002714 (PMC3359971; doi:10.1371/journal.pgen.1002714)
Supplement: Table S1 — Effect estimates for significant interactions between SNPs surrounding HMGCR and LIPC on HDL-C in EA, AA, and HA cohorts. (DOC) [file pgen.1002714.s007.doc]

**Table S1**. **Effect estimates for significant interactions between SNPs surrounding *HMGCR* and *LIPC* on HDL-C in EA, AA, and HA cohorts**

| Test Stage | Cohort^a^ | SNP 1 | SNP 2 | Interaction terms and *P*-values | | | | | | | | R-square | |
| --- | --- | --- | --- | --- | --- | --- | --- | --- | --- | --- | --- | --- | --- |
|  |  |  |  | $i_{aa}$ | *P* | $i_{ad}$ | *P* | $i_{da}$ | *P* | $i_{dd}$ | *P* | Marginal^b^ | Interaction^c^ |
| Discovery | ARIC EA | rs12916 | rs1532085 | -0.65 | 0.05 | -0.84 | 0.06 | 1.45 | 1.3x10^-3^ | 2.28 | 2.3x10^-4^ | 0.5% | 0.2% |
| Fine Mapping | ARIC EA | rs3846662 | rs2043085 | -0.84 | 8.1x10^-3^ | -1.30 | 2.6x10^-3^ | 1.68 | 1.3x10^-4^ | 1.75 | 3.5x10^-3^ | 0.5% | 0.3% |
| Validation | MESA EA | rs3846662 | rs1973688 | 1.24 | 0.089 | 1.31 | 0.17 | 4.84 | 1.4x10^-6^ | 3.96 | 2.8x10^-3^ | 0.2% | 0.9% |
| Validation | FHS EA | rs55727654 | rs473422 | 2.82 | 0.017 | -1.37 | 0.38 | -0.53 | 0.69 | -0.98 | 0.58 | 0.4% | 0.5% |
| Validation | MESA HA | rs1423527 | rs7163280 | -1.69 | 0.047 | -2.92 | 7.6x10^-3^ | 3.65 | 1.5x10^-3^ | 0.40 | 0.78 | 0.5% | 1.1% |
| Validation | ARIC AA | rs3761743 | rs567838 | 2.34 | 0.031 | 0.06 | 0.96 | -1.42 | 0.34 | -4.36 | 0.015 | 0.2% | 1.0% |

^a^ EA denotes European American; HA denotes Hispanic American; AA denotes African American.

^b^ Difference in R-squares between two models with or without the marginal effects (additive and dominance) of the two SNPs individually, which quantifies the fraction of overall variance in HDL-C level that is marginally explained by the two SNPs.

^c^ Difference in R-squares between two models with or without the four interaction terms, which quantifies the fraction of overall variance in HDL-C level that is explained by the interaction terms alone.
Note: Lines in this table correspond to Table 1 in main text.
